# Case Report: Adrenal schwannoma associated with ganglioneuroma

**DOI:** 10.3389/fsurg.2024.1416801

**Published:** 2024-09-19

**Authors:** Dong-Lin He, Run-lin Feng, Jia-gui Chai, Xin Guo, Chang-xing Ke

**Affiliations:** ^1^ Department of Urology, The Second Affiliated Hospital of Kunming Medical University, Kunming, China; ^2^ Department of Pathology, The Second Affiliated Hospital of Kunming Medical University, Kunming, China

**Keywords:** adrenal gland, schwannoma, ganglioneuroma, adrenal tumors, collision tumors

## Abstract

**Background:**

An adrenal collision tumor (ACT) denotes the presence of distinct tumors with diverse behavioral, genetic, and histological features independently co-existing within the adrenal tissue without intermingling, and occurrences of such cases are infrequent. The concurrent occurrence of adrenal schwannoma and adrenal ganglioneuroma is exceedingly rare, and the diagnosis of these ACTs has been notably challenging due to their atypical clinical manifestations and imaging characteristics.

**Case summary:**

A 37-year-old man presented to the hospital 3 weeks after a computed tomography (CT) examination that revealed a left adrenal mass. Physical examination findings were unremarkable. Both CT and magnetic resonance imaging scans indicated the presence of a left adrenal mass. Plasma cortisol, adrenocorticotropic hormone, and renin–angiotensin–aldosterone system tests yielded normal results. Preoperative imaging confirmed the diagnosis of left adrenal pheochromocytoma. After thorough surgical preparation, a laparoscopic partial left adrenalectomy was performed. Subsequent postoperative pathological analysis identified adrenal schwannoma in conjunction with adrenal ganglioneuroma. The patient recovered well and was discharged on postoperative day 4. A routine urology clinic visit was included in his postoperative care plan. During follow-up assessments, CT scans of the left adrenal gland revealed no abnormalities.

**Conclusion:**

Adrenal schwannoma combined with ganglioneuroma represents an exceptionally rare collision tumor characterized by the absence of typical clinical or imaging features, leading to potential misdiagnosis. Adrenal incidentalomas present as multifaceted conditions, and this case serves to heighten awareness of their intricate nature. Due to the challenges in preoperative differentiation of various adrenal mass types, postoperative pathological analysis is imperative for guiding the subsequent treatment course for the patient.

## Introduction

Adrenal schwannoma and ganglioneuroma (GN) represent rare incidentalomas of the adrenal gland, typically discovered fortuitously on imaging studies. Clinical manifestations are often atypical and asymptomatic (1). However, the mass effect can manifest as epigastric and/or celiac pain (2–4). Furthermore, accurate imaging diagnosis is challenging due to the lack of specific radiological features, complicating the diagnostic process (5, 6). Here, we present a case of combined adrenal schwannoma and ganglioneuroma to enhance our comprehension of the diagnosis and management of this condition.

## Case introduction

**Figure 1:**
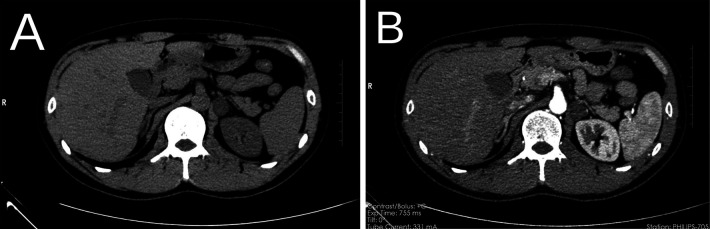
Preoperative CT, left retroperitoneal mass, plain CT **(A)**, contrast-enhanced CT scan **(B)**. CT, computed tomography.

A 37-year-old man was admitted to the hospital after the discovery of a 3-week-old adrenal mass during a computed tomography (CT) examination. The patient did not exhibit symptoms such as hypertension or hypokalemia. There was no significant medical history available, and the patient denied any personal or familial association with cancer. Physical examination revealed the absence of a palpable mass or tenderness upon percussion in the bilateral renal region. Biochemical tests showed unremarkable results: adrenocorticotropic hormone (ACTH) level of 61.56 pg/ml (normal range 7.2–63.4); cortisol level of 28.51 pg/ml (normal range 4.26–24.85); and an aldosterone to renin ratio (ARR) of 22.75. The CT scan with contrast revealed a 2.0 cm × 1.8 cm × 2.4 cm nodule in the left retroperitoneum, displaying inhomogeneous enhancement, the mean Hounsfield unit (HU) before enhancement was 12.9 HU, while the value subsequent to enhancement was 39.2 HU (Figure 1). Magnetic resonance imaging (MRI) with contrast delineated a retroperitoneal mass characterized by a well-defined border, varying internal signal intensity, slightly low signal on T1-weighted imaging, slightly high signal on T2-weighted imaging, and significant enhancement after contrast administration. No enlarged lymph nodes were identified in the retroperitoneal region. Based on imaging findings, a pheochromocytoma was suspected among the differential diagnoses of adrenal tumors, including adrenal adenoma, myelolipoma, lipoma, adrenal carcinoma, and metastatic carcinoma. After a detailed discussion with the patient, the decision was made to surgically remove the mass.

**Figure 2:**
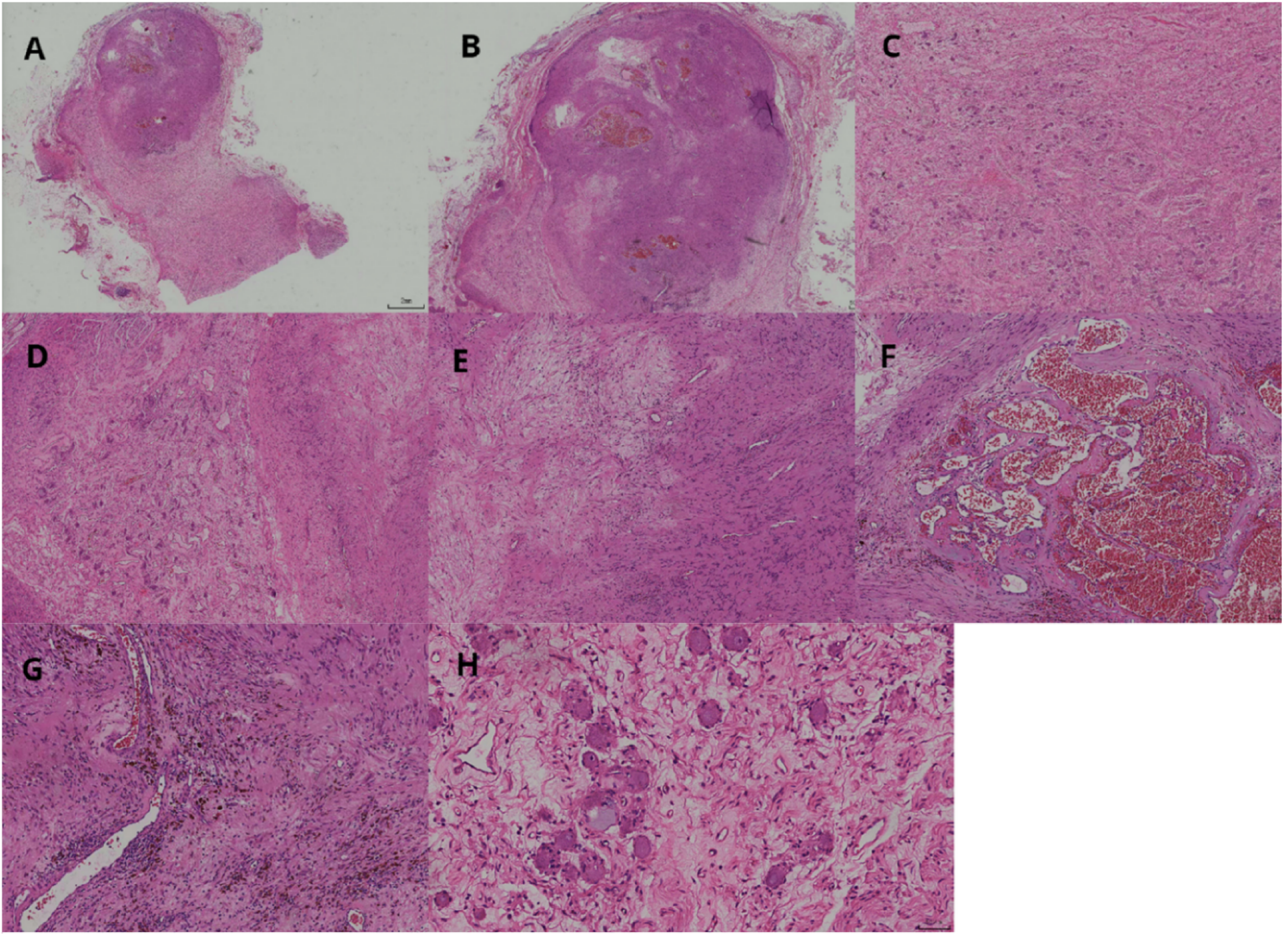
Two tumor components visible at low magnification **(A)**. Schwannoma lesions in the form of round-like nodules **(B)**. Ganglioneuroma lesions as diffuse lamellar structures **(C)**. Well-demarcated nodular cell tumor (left side) and nerve sheath tumor (right side) **(D)**. Schwannoma consists of sparse and dense areas **(E)**. Dilated vascular plexus visible within the sparse area of the schwannoma, resembling cavernous hemangioma structure **(F)**. Mucinous metaplastic lipid cells and hemosiderin-containing components are seen in the tumor of schwannoma **(G)**. Ganglion cells have abundant cytoplasm and large, round, or oval nuclei with 1–3 nucleoli **(H)**.

**Figure 3:**
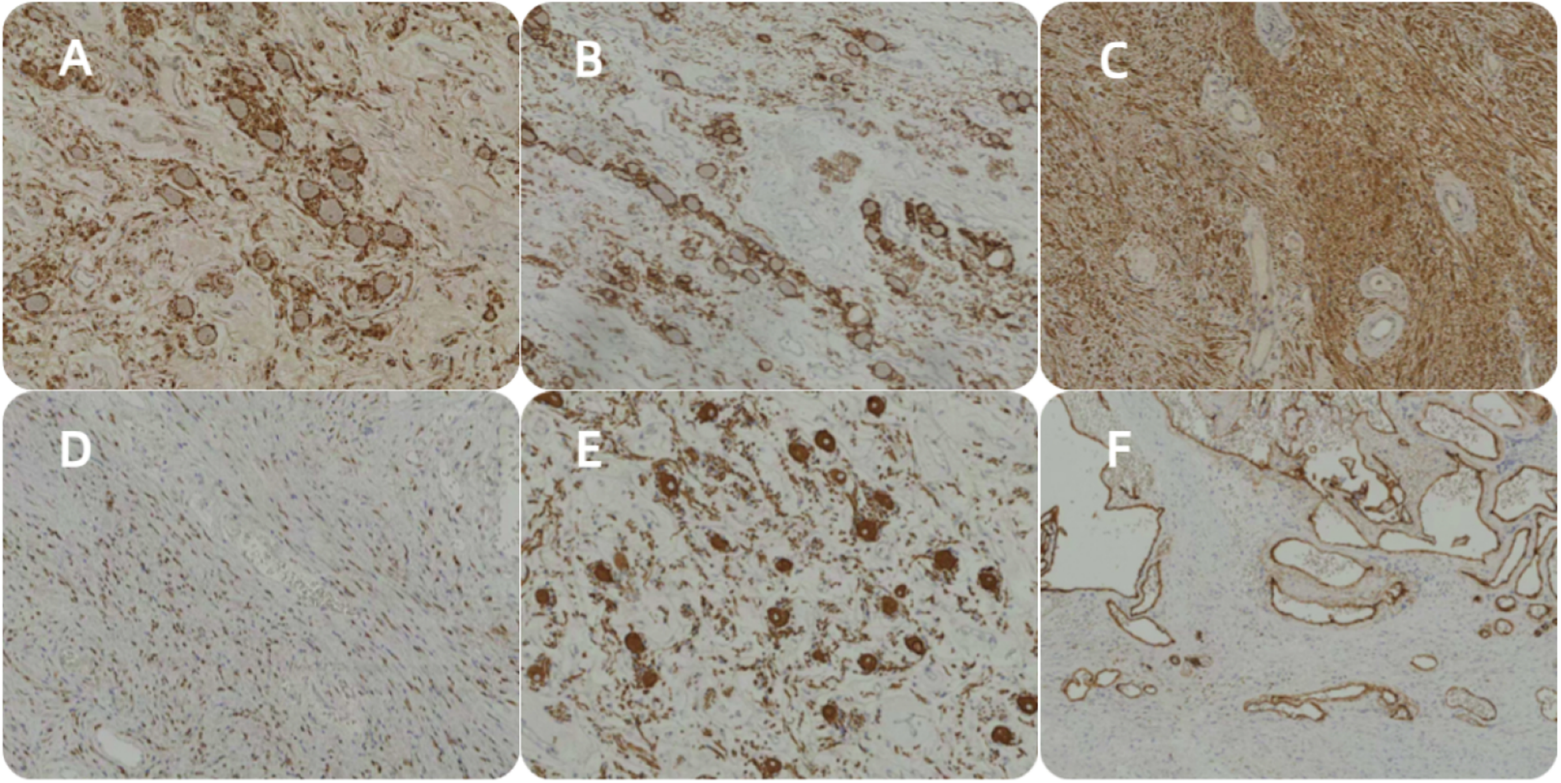
Ganglioneuroma S100(+), ganglioneuroma CD56(+), schwannoma S100(+), schwannoma SOX10(+), ganglioneuroma NF(+), and schwannoma CD34(+) **(A**–**F)**.

After appropriate surgical preparation, which involved preoperative administration of oral phenoxybenzamine hydrochloride, a laparoscopic partial left adrenalectomy was conducted. The mass was meticulously dissected and extracted intact. A visual inspection revealed a grayish-white and grayish-brown mass enclosed in an intact capsule, measuring approximately 3 cm × 2 cm × 1.5 cm and displaying a cystic appearance upon sectioning. Pathological assessment confirmed the presence of adrenal ganglioneuroma combined with schwannoma (Figures 2, 3).

Fortunately, the patient made a remarkable recovery and was discharged from the hospital 4 days postoperatively. A follow-up appointment with the urology clinic was included in his treatment regimen. To date, no abnormalities in the left adrenal gland have been identified on CT imaging during the follow-up period. One limitation of this study is the lack of refinement of preoperative catecholamine testing.

## Related literature learning

Adrenal schwannoma and adrenal ganglioneuroma are both uncommon adrenal neoplasms, representing approximately 0.2%–0.5% and 0.2%–1.6% of all adrenal tumors, respectively, according to population-based studies (2). Adrenal collision tumors are a rare phenomenon, characterized by the presence of two histologically distinct tumors within the same adrenal gland, exhibiting clear demarcation without significant tissue intermingling. In contrast, adrenal composite tumors involve tumors of two distinct origins concurrently arising in the adrenal gland, displaying notable tissue intermixing (7). Adrenal collision tumors, comprising adrenal ganglioneuroma and schwannoma, are exceedingly rare, with only one reported case to date, to the best of our knowledge (8).

This case shares both similarities to and differences from the one documented by Porter et al. (8). In that case, a female patient with a cervical cancer history exhibited symptoms suggestive of a hormone-secreting tumor. Conversely, our case involved a patient devoid of personal or familial tumor history and who was asymptomatic. Imaging revealed a smaller mass devoid of significant central necrosis. The postoperative pathology findings were akin to those reported by Porter et al. However, our surgical approach differed, as we opted for laparoscopic partial adrenalectomy (LPA) with preservation of the affected adrenal gland, yielding no anomalies during subsequent follow-up assessments. Both cases underscore the challenges associated with a preoperative diagnosis and the intricacies of adrenal gland tumor management.

Abdominal tumors of neurogenic etiology can be categorized based on their origin within the nervous system. These classifications encompass tumors arising from ganglion cells [such as ganglioneuroma, ganglion-neuroblastoma (NB), and neuroblastoma], originating from the paraganglionic system (including pheochromocytoma and paraganglioma), or derived from nerve sheaths [such as schwannoma, neurofibroma, neurofibromatosis, and malignant peripheral nerve sheath tumor (MPNST)] (9). Tumors originating from ganglion cells can be subcategorized based on their cellular composition. Tumors predominantly comprising neuroblastoma cells are recognized as NB, whereas those composed solely of mature ganglion cells and other mature tissues are termed GN. Tumors exhibiting a mixture of immature and mature cell types are classified as ganglioneuroblastoma (GNB). GNB and GN tumors are malignant or possess malignant potential and are prevalent malignant neoplasms in pediatric patients, whereas GN is considered a benign tumor (10). Within the paraganglionic system, tumors originating from chromaffin cells in the adrenal medulla are identified as pheochromocytomas. Conversely, tumors developing in other regions of the paraganglia are termed paragangliomas, which exhibit a higher degree of aggressiveness compared to pheochromocytomas (9). In tumors of neural sheath origin, schwannoma is a benign neoplasm originating from nerve sheath cells known as Schwann cells. Neurofibroma, another benign tumor, comprises tumorous Schwann cells but also contains a non-neoplastic component. Neurofibromatosis encompasses a spectrum of disorders, including two distinct types: NF-1 and neurofibromatosis type 2. MPNSTs are aggressive spindle-cell tumors of neural origin that arise within pre-existing Schwann cell tumors, predominantly within plexiform neurofibromas (11).

Adrenal schwannoma is an uncommon tumor originating from Schwann cells of the peripheral nervous system, which form the myelin sheath around nerve fibers. While schwannomas can arise in various body locations, they most commonly affect the head, neck, and the surfaces of flexor muscles in the limbs. Involvement of the adrenal gland is exceptionally rare (4, 12). Although cases have been documented across all age groups, the incidence appears to be highest among middle-aged individuals, with a higher prevalence noted in women (13). The majority of adrenal schwannomas are benign, non-hormonal, and associated with a favorable prognosis (14). Histologically, adrenal schwannomas exhibit a high cellular region consisting of spindle-shaped Schwann cells (Antoni A zone) and a less densely organized low cellular region (Antoni B zone). Neural sheath tumors are classified based on their growth patterns, including conventional, ancient, cellular, plexiform, epithelial, and microcystic types (13, 14). Immunostaining demonstrates strong and diffuse reactivity to S-100 protein in schwannomas (15). On abdominal ultrasound, adrenal schwannomas typically present as well-defined, homogeneous, hypoechoic masses, although mixed echoes may be observed secondary to degenerative alterations. Non-enhanced CT scans of adrenal schwannomas show a mean attenuation of 30 HU, with enhancement after intravenous contrast administration increasing to an average of 60 HU (16). MRI demonstrates low signal intensity on T1-weighted images and heterogeneous high signal intensity on T2-weighted images for adrenal schwannomas (12).

Adrenal ganglioneuroma, another uncommon neurogenic tumor, is a benign neoplasm originating from neural crest cells and characterized by a stroma comprising mature Schwann cells, ganglion cells, and nerve fibers. Like adrenal schwannoma, adrenal ganglioneuroma most frequently occurs in middle-aged individuals, with a higher prevalence among women (6). These tumors typically manifest on the right side of the body, are commonly asymptomatic, hormone non-secreting, and associated with a favorable prognosis (17). The prognosis for adrenal ganglioneuroma is generally favorable. On ultrasound imaging, adrenal ganglioneuromas typically present as solid, well-demarcated masses with a homogeneous and hypoechoic appearance (3). When visualized on CT scans, these tumors appear as solid, homogeneous, hypoattenuating lesions with well-defined borders. Adrenal ganglioneuromas exhibit low attenuation on CT imaging, averaging approximately 30–35 HU, and demonstrate slow, low to mildly heterogeneous enhancement after contrast administration. MRI findings for ganglioneuromas show low signal intensity on T1-weighted images and variably high signal intensity on T2-weighted images (2). A notable characteristic of adrenal ganglioneuromas is their spiral appearance, comprising thicker Schwann cells and intertwined collagen fibers (16, 18).

Surgical intervention, particularly adrenalectomy, remains the cornerstone of treatment for these benign neurogenic tumors, with the majority of cases managed through surgical resection (2). The introduction of minimally invasive surgical approaches has notably enhanced treatment options, with laparoscopic total adrenalectomy (LTA) emerging as the preferred choice for managing adrenal masses. This preference is primarily attributed to the advantages it offers, including reduced postoperative complications and quicker recovery times compared to traditional open surgery. The increasing adoption of minimally invasive techniques has also led to the more frequent utilization of LPA aimed at preserving adrenal gland function in clinical practice (19). LPA was initially utilized in the management of patients with familial bilateral pheochromocytomas to significantly reduce the risk of adrenal insufficiency after bilateral adrenalectomy and mitigate complications associated with prolonged postoperative steroid hormone administration (20). In recent times, LPA has been increasingly employed in the treatment of unilateral benign adrenal masses with intact contralateral adrenal glands, resulting in favorable outcomes (21). Kaye et al. advocated for partial adrenalectomy as the preferred primary treatment approach for small adrenal masses (22). Research on incidental adrenal tumors has revealed a relationship between tumor size and malignancy, suggesting a critical threshold of 4 cm that distinguishes adrenal carcinoma from other tumor types (23). This implies that LPA may be a suitable treatment option for adrenal masses measuring less than 4 cm, especially in the absence of malignant characteristics. Nevertheless, the utilization of LPA in individuals with unaffected contralateral adrenal glands remains a subject of ongoing discussion, and this case will contribute valuable data to support further investigations in this area.

## Conclusion

The co-occurrence of ganglioneuroma and schwannoma within the adrenal gland represents an exceptionally rare occurrence. Clinical diagnosis poses significant challenges, with potential for misdiagnosis even with the aid of imaging modalities. Consequently, postoperative pathological evaluation is essential for accurate diagnosis. Surgical resection remains the cornerstone of treatment for these tumors, with a favorable prognosis observed postoperatively. Given the rarity of this presentation, the importance of conducting long-term follow-up studies cannot be overstated. These investigations will provide valuable insights into the prognosis and potential long-term recurrence patterns associated with this unique tumor entity. Consequently, ongoing monitoring of the patient will be maintained, and any pertinent updates regarding changes in the patient's condition will be documented in subsequent reports.

## Data Availability

The original contributions presented in the study are included in the article/Supplementary Material, further inquiries can be directed to the corresponding authors.
